# Provision of Surgical Services to COVID-19-Infected Patients at a Tertiary Care Center in Pakistan: A One-Year Clinical Review of the Year 2020 in General Surgery Department

**DOI:** 10.7759/cureus.12705

**Published:** 2021-01-14

**Authors:** Osama Ahmed, Muhammad Sohaib Asghar, Muhammad Nadeem Khurshaidi, Farah Yasmin, Noureen Kanwal, Afsheen Javaid Khokher, Asma Tariq, Najia Mallick, Rabail Yaseen, Maira Hassan

**Affiliations:** 1 General Surgery, Liaquat National Hospital, Karachi, PAK; 2 Internal Medicine, Dow University of Health Sciences, Karachi, PAK; 3 General Surgery, Dow University of Health Sciences, Karachi, PAK; 4 Internal Medicine, Liaquat National Hospital, Karachi, PAK

**Keywords:** surgery, infectious disease, infectious disease control, covid-19, emergent general surgery, covid-19 in pakistan, elective surgical procedures, clinical audit, retrospective research, intervention

## Abstract

Background and objectives

The frequency of COVID-19-positive or suspicious patients grew steadily, and these patients were received in emergency and outpatient departments at an unprecedented pace for the need of an elective or emergent surgical assessment. We conducted this survey to document the number of surgeries performed on COVID-19-positive patients during the ongoing pandemic at a tertiary care center in Pakistan.

Materials and methods

A retrospective clinical audit was conducted in a tertiary care hospital that receives surgical cases from almost all over the country. Ethical approval was granted prior to the execution of this intra-departmental audit. Both patients who were admitted to general surgery and visited on a consultative basis in other departments during the year 2020 were evaluated, and only those having COVID-19 polymerase chain reaction (PCR)-positive were included. Those with PCR-negative were omitted from the analysis. All the surgical procedures performed in these patients, along with those managed conservatively, were analyzed. Basic and demographic data of all patients were collected from electronic medical records. The data were defined as either mean and standard deviation or frequency and relative percentages. The normality of the data was verified by the Shapiro-Wilk test. Parametric analysis was used to interpret the disparity in descriptive statistics. Although the categorical results were compared by cross-tabulation, the degrees of significance were calculated either by chi-square test or Fisher's exact test according to the distribution of the data. A p value of less than 0.05 was considered significant (two-tailed).

Results

A total of 79 COVID-19-positive patients were provided with surgical services and subsequently analyzed. The mean age of those patients was 48.88 ± 16.62 years. The mean length of stay in the hospital was 2.10 ± 3.52 with indifference among gender and mode of treatment (either surgical or conservative). The study participants were 59.5% males and 40.5% females, and only 6.3% had a past surgical history. Most patients were admitted through the outpatient department (65.8%), and only a few were referrals from other departments (10.1%); 64.5% of patients were managed in general wards, 24.0% in critical care units, and 11.4% in intensive care units. Surgical intervention was done in 60.8% of the COVID-19-positive patients, while the rest 39.2% were conservatively managed. Among whom, 63.3% were discharged, 29.1% of them left against medical advice (LAMA), with a 7.6% death rate during the hospital stay. The frequent comorbidities were diabetes (27.8%) and hypertension (26.6%), although most patients had no comorbidities (49.3%). Symptomatic gall stones were the most frequent reason for surgical admission in COVID-19-positive patients, while the most frequent surgical intervention performed was laparoscopic cholecystectomy. Males were comparatively managed more frequently by surgical intervention and females been more conservatively managed (p = 0.037). Out of the six mortalities, five were surgically managed. Seventy seven percent of the surgically managed patients were discharged, and the majority of LAMA patients were being conservatively managed (p < 0.001).

Conclusion

This study was done to analyze the demographic factors associated with the outcomes of surgical interventions performed on COVID-19-positive patients.

## Introduction

The first case of the peculiar virus was detected in Wuhan, China, in December 2019, and initially it was treated as pneumonia of unknown etiology. After analyzing the samples, a novel virus was detected, and it was declared novel coronavirus pneumonia [1,2]. Later the name was given to this virus as severe acute respiratory syndrome coronavirus-2 (SARS-CoV-2) by the International Committee on Taxonomy of Virus [3]. The World Health Organization (WHO) on February 11, 2020, named this virus “Coronavirus disease-19 (COVID-19)" [4].

The first COVID-19 case was diagnosed in Pakistan on February 26, 2020, in the city of Karachi. Later on, it became impossible to control the spread of infection and soon it spread to the whole of Pakistan. With the increasing rate of incidence of disease, the number of patients with the COVID-19 disease or suspected of having the disease increased rapidly, and these patients were being received in emergencies and outpatient departments at an alarming rate due to which decision was made to postpone elective surgeries [5]. However, it was not possible to postpone emergency surgeries, and thus, healthcare professionals were at high risk of exposure to the disease and infection. The extensive spread of this disease caused the hospitals to continue prioritizing treatment of COVID-19 patients and deal with emergency surgeries only so as to reduce chances of mortality while postponing elective surgeries if possible.

The purpose of this study was to analyze the surgical procedures of COVID-19-positive patients in the general surgery department performed during the ongoing pandemic in a tertiary care hospital.

## Materials and methods

A retrospective clinical audit was conducted in a tertiary care hospital that receives surgical cases from almost all over the country. Ethical approval was granted prior to the execution of this intra-departmental audit. Both patients who were admitted to general surgery and visited on a consultative basis in other departments during the year 2020 were evaluated, and only those having COVID-19 polymerase chain reaction (PCR)-positive were included. Those with PCR-negative were omitted from the analysis. All the surgical procedures performed in these patients, along with those managed conservatively, were analyzed. Basic and demographic data of all patients were collected from electronic medical records. The data was assembled into Statistical Package for Social Sciences (SPSS, version 25.0) for Windows (IBM Corp., Armonk, NY). The data were defined as either mean and standard deviation or frequency and relative percentages. The normality of the data was verified by the Shapiro-Wilk test. Parametric analysis was used to interpret the disparity in descriptive statistics. Although the categorical results were compared by cross-tabulation, the degrees of significance were calculated either by chi-square test or Fisher's exact test according to the distribution of the data. A p value of less than 0.05 was considered significant (two-tailed).

## Results

A total of 79 COVID-19-positive patients were provided with surgical services and subsequently analyzed. The mean age of those patients was 48.88 ± 16.62 years. The mean length of stay in the hospital was 2.10 ± 3.52 with indifference among gender and mode of treatment (either surgical or conservative). The study participants were 59.5% males and 40.5% females, and only 6.3% had a past surgical history. Most patients were admitted through the outpatient department (65.8%), and only a few were referrals from other departments (10.1%); 64.5% of patients were managed in general wards, 24.0% in high-dependency units, and 11.4% in intensive care units. Surgical intervention was done in 60.8% of the COVID-19-positive patients, while the rest 39.2% were conservatively managed. Among whom, 63.3% were discharged, 29.1% of them left against medical advice (LAMA), and 7.6% were expired during the hospital stay. The frequent comorbidities were diabetes (27.8%) and hypertension (26.6%), although most patients were having no comorbidities (49.3%). Symptomatic gall stones were the most frequent reason for surgical admission in COVID-19-positive patients, while the most frequent surgical intervention performed was laparoscopic cholecystectomy as shown in Table 1.

**Table 1:** Demographic data of the study population (n = 79). HDU: High-dependency unit; ICU: intensive care unit; LAMA: left against medical advice.

Variables	Descriptives/Frequency
Mean age (in years)	48.88 ± 16.62
Consultation	Primary: 71 (89.9%); Referral: 8 (10.1%)
Mode of admission	Emergency: 27 (34.2%); Outpatient department: 52 (65.8%)
Length of stay (in days)	2.10 ± 3.52
Gender	Males: 47 (59.5%); Females: 32 (40.5%)
Past surgical history	Present: 5 (6.3%); Absent: 74 (93.7%)
Hospital stay	Ward: 51 (64.5%); HDU: 19 (24.0%); ICU: 9 (11.4%)
Outcome	Discharged: 50 (63.3%); Expired: 6 (7.6%); LAMA: 23 (29.1%)
Treatment	Surgical intervention: 48 (60.8%); Conservative: 31 (39.2%)
Comorbidities	Diabetes: 22 (27.8%)
	Hypertension: 21 (26.6%)
	Ischemic heart disease: 4 (5.0%)
	Chronic obstructive pulmonary disease: 2 (2.5%)
	Hypothyroidism: 1 (1.2%)
	Gout: 1 (1.2%)
	Malignancy: 1 (1.2%)
	No comorbidities: 39 (49.3%)
Reason for surgery consultation	Abdominal wound dehiscence: 1 (1.2%)
	Acute cholecystitis: 11 (5.3%)
	Acute appendicitis: 3 (6.0%)
	Bomb blast injury: 2 (2.5%)
	Breast lump: 2 (2.5%)
	Burst abdomen + Enterocutaneous fistula: 1 (1.2%)
	Carcinoma of anorectum: 1 (1.2%)
	Carcinoma of cheek: 2 (2.5%)
	Esophageal carcinoma: 1 (1.2%)
	Gall bladder perforation: 1 (1.2%)
	Carbuncle: 1 (1.2%)
	Choledocholithiasis: 3 (3.8%)
	Diabetic foot: 3 (3.8%)
	Diaphragmatic rupture: 1 (1.2%)
	Duodenal perforation: 1 (1.2%)
	Obstructed incisional hernia: 2 (2.5%)
	Fistula in ano: 2 (2.5%)
	Symptomatic gallstones: 11 (13.9%)
	Flap necrosis: 5 (2.5%)
	Cellulitis: 3 (3.8%)
	Adenocarcinoma of descending colon: 1 (1.2%)
	Hemosuccus pancreaticus: 1 (1.2%)
	Hypersplenism: 1 (1.2%)
	Inguinal hernia: 2 (2.5%)
	Insulin site abscess: 1 (1.2%)
	Ischemic limb: 1 (1.2%)
	Gluteal abscess: 1 (1.2%)
	Mesenteric ischemia: 2 (2.5%)
	Small bowel obstruction: 1 (1.2%)
	Large bowel obstruction: 1 (1.2%)
	Necrotizing fasciitis: 2 (2.5%)
	Perianal abscess: 3 (3.8%)
	Small bowel perforation: 1 (1.2%)
	Surgical site infection: 1 (1.2%)
	Strangulated inguinal hernia: 2 (2.5%)
	Psoas abscess: 1 (1.2%)
Surgical intervention performed (n = 48)	Above-knee amputation: 1 (1.2%)
	Diversion colostomy: 1 (1.2%)
	Wound debridement: 4 (5.1%)
	Reversal of Hartmann's procedure: 1 (1.2%)
	Ray amputation: 1 (1.2%)
	Pigtail insertion: 1 (1.2%)
	Percutaneous cholecystotomy: 3 (3.8%)
	Open inguinal hernia mesh repair: 1 (1.2%)
	Open appendectomy: 1 (1.2%)
	Mastectomy: 1 (1.2%)
	Lumpectomy: 1 (1.2%)
	Laparoscopic cholecystectomy: 12 (15.2%)
	Incision and drainage: 3 (3.8%)
	Ileostomy reversal: 1 (1.2%)
	Laparoscopic hernia mesh repair: 1 (1.2%)
	Fistulotomy: 1 (1.2%)
	Feeding jejunostomy: 2 (2.5%)
	Exploratory laparotomy: 9 (11.4%)
	Esophagectomy: 1 (1.2%)
	Endoscopic retrograde cholangiopancreatography: 1 (1.2%)
	Angioembolization: 1 (1.2%)

**Figure 1:**
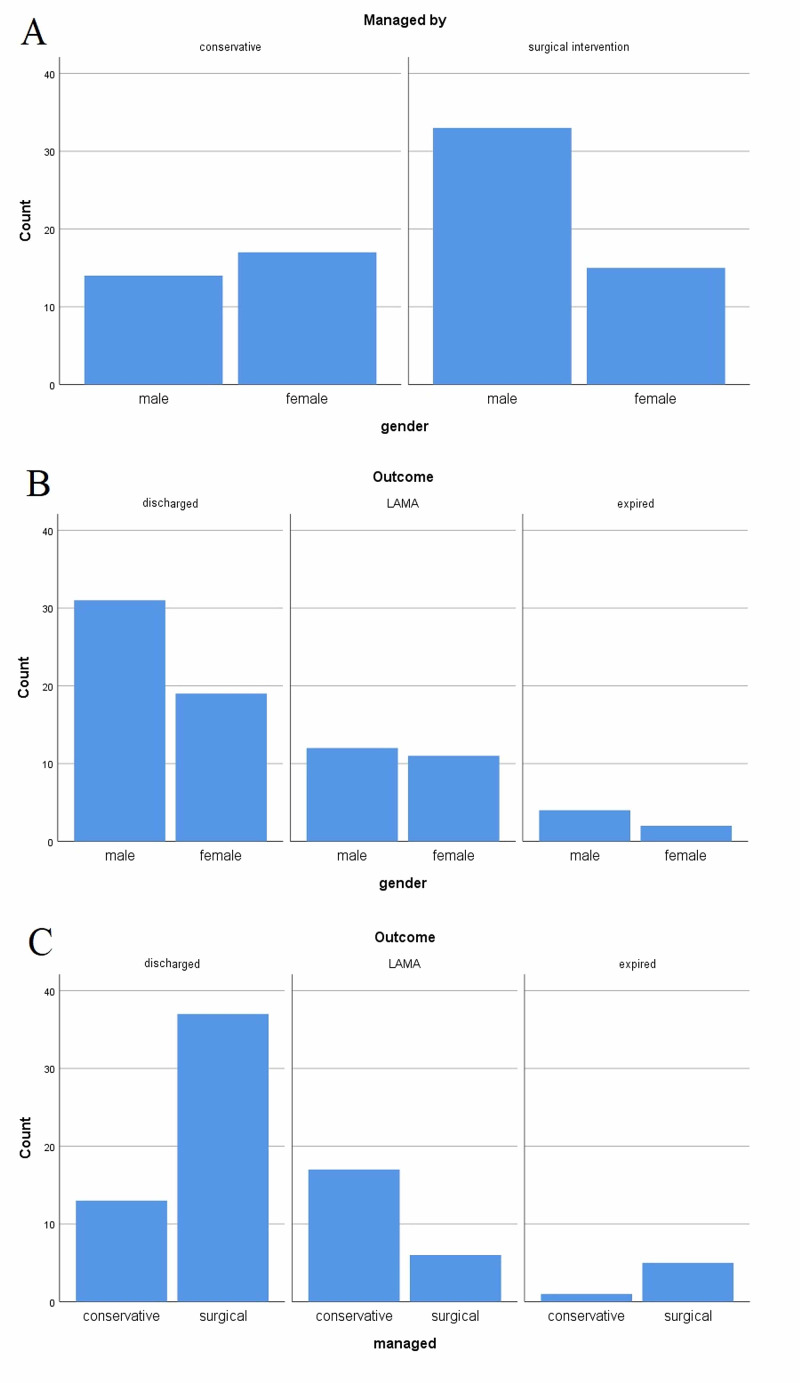
Cross-tabulations of gender with mode of management (A), gender with outcomes (B), and mode of management with outcomes (C). LAMA: Left against medical advice.

Table 2 has shown cross-tabulations of factors analyzed between conservative and surgically managed patients along with their outcomes. It has shown that males were comparatively managed more frequently by surgical intervention and females been more conservatively managed (p = 0.037). Out of the six mortalities, five were surgically managed. Seventy seven percent of the surgically managed patients were discharged, and the majority of LAMA patients were being conservatively managed (p < 0.001) as shown in Figure 1.

**Table 2:** Analyzing factors and cross-tabulation for surgical versus conservative management and outcomes of the study population (n = 79). * indicates p value calculated by independent sample t-test; ** indicates chi-square test; and † indicates Fisher’s exact test. LAMA: Left against medical advice.

Variables	Factors		p value
Mean age	Males: 50.29 ± 17.57	Females: 46.81 ± 15.16	0.364*
	Conservative: 48.93 ± 19.68	Surgical: 48.85 ± 14.53	0.984*
Length of stay	Males: 1.97 ± 2.15	Females: 2.28 ± 4.93	0.711*
	Conservative: 1.70 ± 3.95	Surgical: 2.35 ± 3.24	0.432*
Conservative management	Males: 14 (29.8%)	Females: 17 (53.1%)	0.037**
Surgical management	Males: 33 (70.2%)	Females: 15 (46.9%)	
Expired	Males: 4 (8.5%)	Females: 2 (6.3%)	0.664^†^
LAMA	Males: 12 (25.5%)	Females: 11 (34.3%)	
Discharged	Males: 31 (66.0%)	Females: 19 (59.4%)	
Expired	Conservative: 1 (3.2%)	Surgical: 5 (10.4%)	<0.001^†^
LAMA	Conservative: 17 (54.8%)	Surgical: 6 (12.5%)	
Discharged	Conservative: 13 (41.9%)	Surgical: 37 (77.1%)	

## Discussion

The whole world is affected by COVID-19 pandemic, and hospitals are flooded by COVID-19-positive patients whether it is an outpatient department or emergency department. This pandemic is spreading faster and faster by the day, affecting all healthcare systems and operations around the globe and Pakistan was no exception. Most hospitals in Pakistan stopped outpatient settings but not our hospital due to a huge influx of patients. The emergency department was the most affected in all of the hospital departments.

We took necessary precautions to safely conduct surgeries with minimal contact amidst the pandemic. Various recommendations have been published by anesthesiologists regarding the equipment and protection required to do intubation safely [5,6]. The ongoing research into the epidemiology, pathophysiology, and treatment regarding the patients infected with COVID-19 and its respective effects on public health for surgical treatment on individuals with suspicion or diagnosis of COVID-19 infection have not been of prime importance [7].

The most number of procedures performed is in the department of general surgery, and the same results were also reported in another study, with their top two primary interventions being gastrointestinal and general surgery [8]. As hospitals restarted elective surgeries, new protocols were made under the influence of studies that were researching COVID-19 infections. In a study, it was stated that some of the people infected with COVID-19 were asymptomatic, and in 78 patients who tested positive for COVID-19 infection, 42% were not showing any symptoms [9]. As further studies were undertaken, it was also suggested that sensitivity and specificity were significant when the patients showed symptoms but got worse when patients are asymptomatic [10,11].

It is an infectious disease, but a considerable amount of COVID-19 patients were asymptomatic. So protocols were setup that any patient undergoing any surgical procedure whether elective or emergency was assumed to be positive until proven otherwise. It was also suggested in studies that the virus can live on contact surfaces for several hours despite being transmitted by droplets [6]. That posed a major risk for healthcare professionals if they happened to come into contact with these surfaces or risked transmitting the virus to their loved ones. Another set of protocols was made to completely disinfect these surfaces, and much effort was made to leave adequate time between two surgeries to disinfect the operating theater rooms properly. Another protocol was followed that both anesthesia and surgical members were reduced to a minimum during induction of patient in the operating theater room.

The use of laparoscopy on patients is still controversial due to its use of gases, and Yu et al. suggested that you cannot ignore the fact that virus can also be transmitted via oro-fecal route [12]; so better management of laparoscopic gases was needed. On the other hand, Morris et al. showed that laparoscopy can be done safely on gynecological procedures as this transmission is yet to be proven [13]. Keeping this in mind, 15% of laparoscopic cholecystectomy was performed and none of the patients developed any complication in our study, and one laparoscopic appendectomy was also performed without any postoperative event. Based on this data we can say that laparoscopic procedures are safe to perform until proven otherwise.

Performing surgeries during a life-threatening viral pandemic has its ethical implications too. The use of gowns, gloves, personal protective equipment, anesthesia, operating theater, and staffing needed extra resources that needed to be reviewed regularly. The goal of our study was to show that as more and more elective surgeries get postponed, this phenomenon will directly affect emergencies as more patients will present in the emergency department. This growing number of positive patients and increasing resumptions of elective cases should not stop us from doing what healthcare professionals do which is managing the patients timely at the expense of workload and surgical planning. The limitations of this study are single-center design, a limited number of patients, retrospective nature, and lack of randomization in the study.

## Conclusions

As elective surgical procedures were halted during the peak of the first wave of COVID-19, our hospital was still performing emergent surgical interventions on COVID-19-positive patients. This study was done to analyze the demographic factors associated with the outcomes of those patients. One factor that was highlighted was a comparatively decreased length of hospital stay for general surgery patients as most of them were referred for home isolation or other COVID-19 isolation centers after undergoing primary surgical management. A substantial number of patients also went LAMA either due to the hesitation of being referred to a COVID isolation unit or other psychosocial factors that were not part of our survey domain.
